# A phosphate starvation‐driven bidirectional promoter as a potential tool for crop improvement and *in vitro* plant biotechnology

**DOI:** 10.1111/pbi.12653

**Published:** 2016-12-27

**Authors:** Oropeza‐Aburto Araceli, Cruz‐Ramírez Alfredo, Mora‐Macías Javier, Herrera‐Estrella Luis

**Affiliations:** ^1^ Metabolic Engineering LaboratoryUnidad de Genómica Avanzada – LANGEBIO CINVESTAVIrapuatoGuanajuatoMexico; ^2^ Molecular and Developmental Complexity LaboratoryUnidad de Genómica Avanzada – LANGEBIO CINVESTAVIrapuatoGuanajuatoMexico

**Keywords:** phosphate starvation, crop improvement, bioengeneering, roots, enhancer

## Abstract

Phosphate (Pi)‐deficient soils are a major limitant factor for crop production in many regions of the world. Despite that plants have innovated several developmental and biochemical strategies to deal with this stress, there are still massive extensions of land which combine several abiotic stresses, including phosphate starvation, that limit their use for plant growth and food production. In several plant species, a genetic programme underlies the biochemical and developmental responses of the organism to cope with low phosphate (Pi) availability. Both protein‐ and miRNA‐coding genes involved in the adaptative response are transcriptionally activated upon Pi starvation. Several of the responsive genes have been identified as transcriptional targets of PHR1, a transcription factor that binds a conserved cis‐element called PHR1‐binding site (P1BS). Our group has previously described and characterized a minimal genetic arrangement that includes two P1BS elements, as a phosphate‐responsive enhancer (*EZ2*). Here, we report the engineering and successful use of a phosphate‐dependent bidirectional promoter, which has been designed and constructed based on the palindromic sequences of the two P1BS elements present in *EZ2*. This bidirectional promoter has a potential use in both plant *in vitro* approaches and in the generation of improved crops adapted to Pi starvation and other abiotic stresses.

## Introduction

One of the major limitations for sustained plant growth in most soils is the scarcity of inorganic phosphate (Pi). A large fraction of Pi in soils is present as diverse organic and inorganic chemical forms that are not readily available for plant uptake. As this macronutrient is needed for the synthesis of vital molecules such as nucleic acids and phospholipids and for important metabolic processes, plants have evolved a complex and multifactorial strategy to adapt and grow in soils with low Pi abundance (Lynch, 1995; Plaxton, 2004; Raghothama, 1999). Such combined strategy involves the action of Pi as a signal for triggering a signalling cascade to respond to the internal and external level of Pi (Lin *et al*., 2009; Shen *et al*., 2011).

Cells of roots and rhizoids are in charge of Pi uptake and are the first organs involved in sensing Pi availability in the rhizosphere. In Pi‐scarce soils, a still unclear signalling pathway is triggered in the most external cell layers of the roots, and in rhizoids, such pathway activates a transcriptional machinery that induces the expression of mRNA and miRNAs that are involved in the myriad of biochemical and morphological mechanisms that allow the plant to: (i) reconfigure the root system architecture (RSA) and release Pi from the organic compounds in the soil, (ii) increase Pi uptake and transport and (iii) recycle Pi from cellular organic sources (Baker *et al*., 2015; Chiou and Lin, 2011; Rouached *et al*., 2010; Vance *et al*., 2003; Williamson *et al*., 2001).

**Figure 1:**
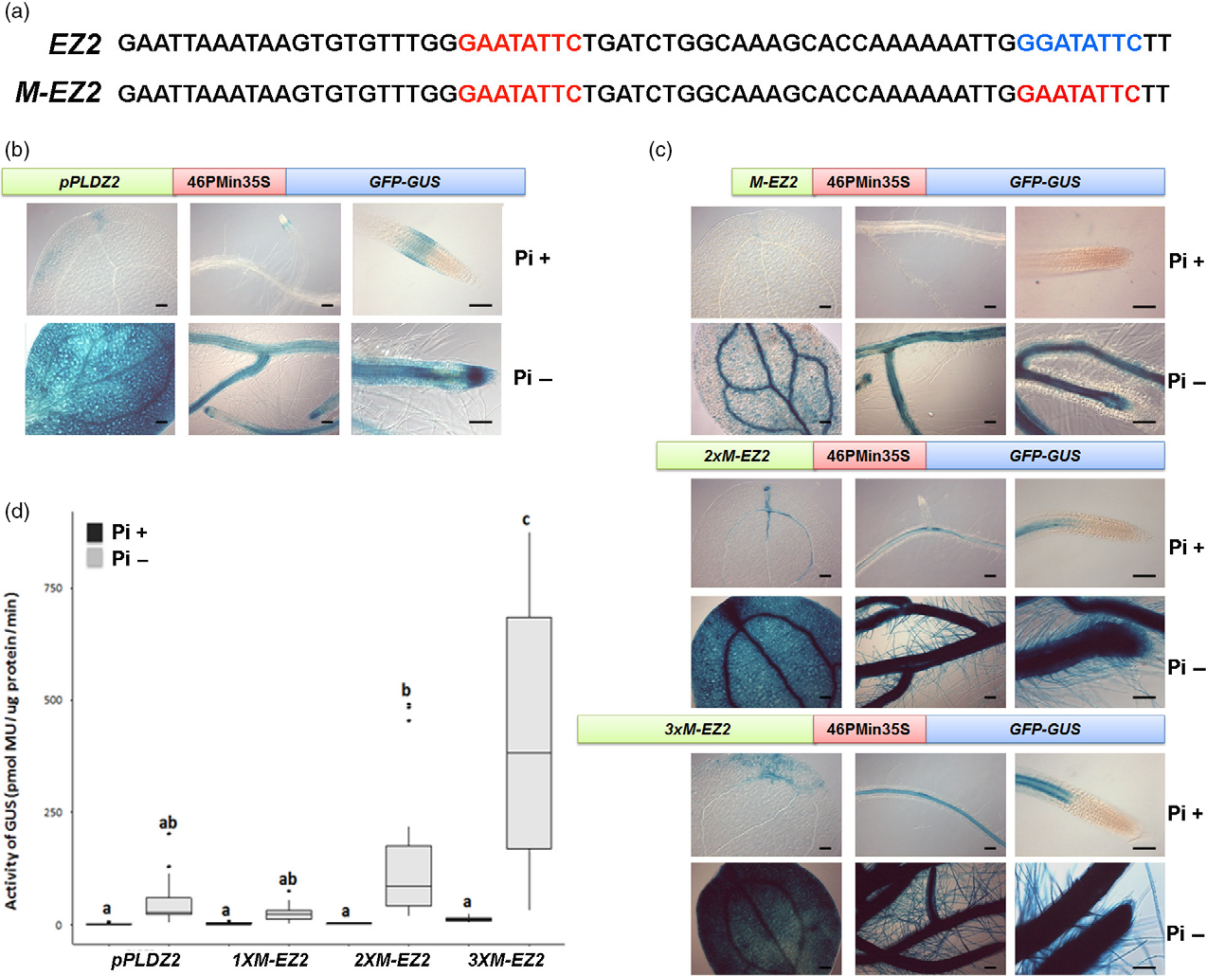
Structure of *EZ2* and *M‐EZ2* and level expression of tandem enhancers. (a) The native upstream sequence of the *PLDZ2* (locus At3g05630) between position −782 and 717 pb relative to the start codon (top sequence), and the modified sequence containing the duplicated P1BS motif (red) to obtain the *M‐EZ2* enhancer (bottom sequence) are shown. (b) Structure and expression of *pPLDZ2‐GUS::GFP*, Arabidopsis plants carrying the *PLDZ2* promoter driving the gene *GUS* were grown in P+ (1 mm) and P− (0 mm) medium for 10 dag. Plants were stained for GUS activity, and cotyledons, lateral roots and root meristematic region were photographed using Nomarsky optics. (c) Structure and expression of chimeric promoters composed of the sequence of modified enhancer once (*1XM‐EZ2*), twice (*2XM‐EZ2*) and three times (*3XM‐EZ2*) inserted upstream of the −46 S minimal 35S promoter driving the expression of *GUS*. Seedlings were grown for 10 dag in medium Pi+ (1 mm) or Pi− (0 mm) and stained for GUS activity. Cotyledons, lateral roots and root apical meristems of representative plants from each line and for each condition (Pi+ and P−) were photographed using Nomarsky optics. Bars, 100 μm. (d) Results of the fluorometric assay of eight independent lines for each construction are shown. Values indicate the mean obtained for two biological replicates and four independent technical replicates for each biological sample. Different letters above bars indicate statistically significant differences based on an ANOVA test.

In Arabidopsis and other plant species, it has been well characterized that under Pi starvation, the expression of several miRNAs and mRNAs involved in the adaptive mechanisms mentioned above is positively regulated by the transcription factor PHOSPHATE STARVATION RESPONSE 1 or PHR1 (Bari *et al*., 2006; Lundmark *et al*., 2010; Nilsson *et al*., 2007; Rubio *et al*., 2001). PHR1 is a MYB‐CC transcription factor with several homologues in diverse plant species, including PSR1 (Phosphate Starvation Response 1), the homolog of PHR1 in the chlorophyte algae *Chlamydomonas reinhardtii* (Wykoff *et al*., 1999). PHR1 homologues share the conserved function of orchestrating the transcriptional programme triggered in response to low Pi availability and activate the transcription of its target genes by binding to P1BS (PHR1‐binding sites), a conserved DNA motif, with the consensus sequence GNATATNC, which is present in the promoters of many phosphate‐responsive genes (Franco‐Zorrilla *et al*., 2004; Müller *et al*., 2007; Sobkowiak *et al*., 2012). We have previously reported the finding and characterization of a conserved enhancer element that regulates the expression of the Arabidopsis *PHOSPHOLIPASE D‐Z2* (*PLDZ2*) gene in response to Pi availability (Oropeza‐Aburto *et al*., 2012). This enhancer element, denominated *EZ2,* is present in the promoter of *PLDZ2* orthologues and in diverse Pi‐responsive genes conserved along plant lineages (Acevedo‐Hernández *et al*., 2012). *EZ*2 is composed by two P1BS motifs that are not identical (sequence of the upstream one is GAATATTC and the other GGATATTC) with a spacer sequence of between 21 and 28 bp in average, depending on the plant species (Figure 1a). It also has a conserved motif in the spacer region with the consensus sequence GCAYCAAA and a motif in its 5′ region with the sequence TTTGG or TTTGC. We modified the *EZ2* native enhancer and found that when the two P1BS elements have the same GAATATTC motif, the induction by Pi starvation is stronger than the native enhancer, indicating that the Arabidopsis PHR1 displays a higher affinity for this Modified *EZ2* (*M‐EZ2*) version of the enhancer (Figure 1a). A recent study demonstrates that when the two P1BS motifs in the promoter of *OsPHF1* were replaced by the same sequence we used in *M‐EZ2*, there is a drastic increase in the affinity of OsPHR2, the PHR1 ortholog in rice, for this type of cis‐regulatory enhancer (Ruan *et al*., 2015).

In this work, we describe the design and functional characterization of a bidirectional promoter based on the *M‐EZ2* enhancer, which responds with high sensitivity to Pi concentrations. Our results show that this novel tool has a potential use in the generation of crops with improved capacity to deal not only with Pi starvation, but also other abiotic stresses associated with Pi‐scarce soils. Moreover, *M‐EZ2*‐based bidirectional promoter can be used to drive gene expression for *in vitro* plant biotechnology, where Pi availability can be used to modulate expression of diverse proteins as reporters and/or resistance markers, in a reversible manner.

## Results

### In‐tandem arrangement of *M‐EZ2*

In a previous work (Oropeza‐Aburto *et al*., 2012), we identified an enhancer element in the regulatory region of the Arabidopsis *PLDZ2* gene. The sequence and arrangement of this enhancer is described in detail in Figure 1a. A modified version of this enhancer when fused to the −46 cauliflower mosaic virus 35S minimal promoter (46PMin35S) was shown to drive high levels of expression of both *UidA* (*GUS*) and *GFP* reporter genes in a low Pi‐dependent manner (Oropeza‐Aburto *et al*., 2012). The modified promoter, named *M‐EZ2,* turns on transcription in response to Pi starvation. To determine whether a promoter with more than one copy of *M‐EZ2* could increase the responsiveness to Pi starvation, we generated synthetic promoters with two or three copies of the *M‐Z2* enhancer *(2XM‐EZ2* and *3XM‐EZ2* versions), fused to the 46PMin35S and a *GUS‐GFP* double reporter gene construct (Figure 1c). Arabidopsis transgenic lines were obtained by transforming plants independently with *1XM‐EZ2‐GUS::GFP*,* 2XM‐EZ2‐GUS::GFP* or *3XM‐EZ2‐GUS::GFP* and its responsiveness to Pi availability tested (Figure 1c).

### The number of *M‐EZ2* copies in the promoter is directly proportional with the strength in *GFP* and *GUS* expression

As a first approach to qualitatively determine the transcriptional effect of different number of *M‐EZ2* copies, seeds from several independent transgenic *1XM‐EZ2‐GUS::GFP*,* 2XM‐EZ2‐GUS::GFP*,* 3XM‐EZ2‐GUS::GFP* and *pPLDZ2‐GUS::GFP* lines were germinated and grown either in P+ (1 mm) or P− (0 mm) medium. Seedlings of 10 days after germination (dag) were stained for GUS activity, and representative images of each line are shown (Figure 1b and c). As previously reported (Oropeza‐Aburto *et al*., 2012), under Pi‐sufficient conditions, *pPLDZ2‐GUS::GFP* seedlings only stain for GUS in the root meristematic region, whereas in Pi‐limiting conditions there is a clear increase in GUS activity in cotyledons and all root tissues (Figure 1b). We observed that for *1XM‐EZ2‐GUS::GFP* lines there is no GUS in Pi+ conditions, while in Pi− conditions *1XM‐EZ2‐GUS::GFP* seedlings showed a similar GUS pattern to that observed for *pPLDZ2‐GUS::GFP* lines grown in P− media, although less intense (Figure 1b and c). Seedlings of *2XM‐EZ2‐GUS::GFP* and *3XM‐EZ2‐GUS::GFP* lines grown in Pi+ conditions showed weak GUS staining in the vascular tissue of both cotyledon and root, but never the root meristematic zone, even when plants were incubated overnight for staining (Figure 1c). However, in Pi− conditions seedlings showed a dramatic increase in GUS activity in all root tissues, including root hairs, a strong was also observed in the cotyledons. In summary, the number of copies of the *M‐Z2* enhancer determines the spatial–temporal patterns of GUS expression and the strength of the response to Pi deprivation (Figure 1c).

To determine in a quantitative manner the increase in GUS expression, we first performed fluorometric GUS assays to quantify the activity of Beta‐Glucuronidase (the protein coded in the *UidA* gene or *GUS*) in seedlings of eight independent lines for each of the synthetic promoters that differ in the number of *M‐EZ2* enhancer elements (Figure 1c–d and Table S1). Seedlings were germinated and grown in medium with phosphate (1 mm) or without phosphate (0 mm) for 10 days after germination (dag) and then GUS activity determined for each line. In P− conditions, the *1XM‐EZ2‐GUS::GFP* seedlings showed an average activity of 25.249 pmol MU/μg protein min, which is only the half of the average value of the full Arabidopsis *PLDZ2* promoter (*pPLDZ2‐GUS::GFP*). The average activity for the *2XM‐EZ2‐GUS::GFP* line was 153.609 pmol MU/μg protein min, which represents 2.9 times higher than full *PLDZ2* promoter, whereas the *3XM‐EZ2‐GUS::GFP* lines showed the highest average value of expression, 433.1 pmol MU/μg protein min, this is 8.321 times more than that showed by the *PLDZ2‐GUS::GFP* lines. Statistical analyses (ANOVA) showed that between the *1XM‐EZ2‐GUS::GFP* and the complete *PLDZ2‐GUS::GFP* there is no significant difference in the level of GUS expression in seedlings grown under Pi‐limiting conditions; by contrast, there is significant differences between the expression directed by the *2XM‐EZ2‐GUS::GFP* and *3XM‐EZ2‐GUS::GFP* synthetic promoters and that observed for the complete *PLDZ2* promoter. Furthermore, in Pi+ media there is no difference in the level of expression directed between the synthetic promoters with that observed for the complete *PLDZ2* promoter (Figure 1d). The fold change of GUS activity between seedling grown in Pi+ media compared to Pi− media for *1XM‐EZ2‐GUS::GFP*,* 2XM‐EZ2‐GUS::GFP* and *3XM‐EZ2‐GUS::GFP* lines was 10.11, 79.44 and 35.01, respectively. The lower fold change induction in *3XM‐EZ2‐GUS::GFP* seedlings with respect to *2XM‐EZ2‐GUS::GFP* is due to a higher basal activity when they are grown in Pi supply media (Table S1). Also in the *3XM‐EZ2‐GUS::GFP* lines in which GUS activity was as high as 848.45 pmol MU/μg protein min, this represents 16.3 times higher than that quantified for the *PLDZ2‐GUS::GFP* line and it was comparable or even higher than the GUS expression observed in constructs driven by the *CAMVS35S* promoter (Table S1).

These results indirectly show that the transcriptional activation in response to Pi availability, as indicated by the level of GUS activity, increases as the number of copies of *M‐EZ2* also increases.

To directly measure the effect of the number of copies of *M‐EZ2* on gene transcription, we performed both semiquantitative reverse transcription PCR (RT‐PCR) and quantitative reverse transcription PCR (qRT‐PCR) assays to determine the levels of *GUS* transcripts. For these experiments, seedlings were germinated and grown for 7 days in Pi+ solid medium (1 mm) and transferred to Pi− liquid medium (0 mm). For RNA extraction, samples were frozen and processed at different time points: 12, 24, 48, 72 and 96 h post‐transference (hpt). Our results showed that *GUS* mRNA levels, when transcription is under the control of the unmodified (native) *EZ2,* were almost absent in seedlings grown in P+ media and a weak gradual response to Pi‐starvation conditions was observed when compared to the RT‐PCR products observed when *GUS* transcription was driven under the control of the full *PLDZ2* promoter and the 1X version of *MEZ2* (Figure 3a). Also, in these RT‐PCR assays we were able to observe that the *2XM‐EZ2* promoter version gradually increases the *GUS* gene transcription upon time in response to Pi starvation in a similar fashion to that observed for the full *PLDZ2* promoter (Figure 3a). Interestingly, the *3XM‐EZ2* promoter version responded earlier and stronger than any other of the promoter versions, including the full *PLDZ2* promoter (Figure 3a).

**Figure 2:**
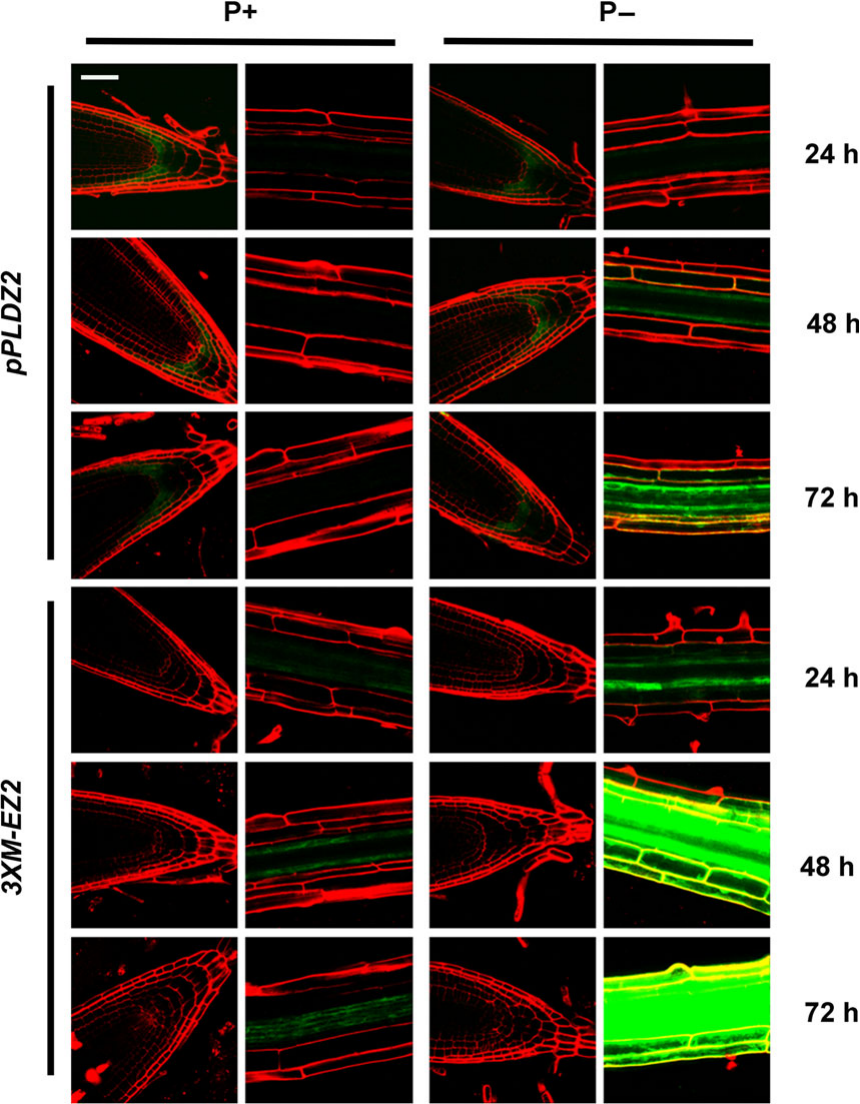
Pi‐starvation‐induced expression of GFP in Arabidopsis plants. Transgenic *pPLDZ2‐GUS::GFP* and *3XM‐EZ2‐GUS::GFP* lines were grown in Pi‐sufficient conditions for 7 dag and induced in Pi‐deficient medium (P−) for 24, 48 and 72 h. Plants grown in P+ medium as control are shown. Photographs of root apical meristem and root elongation zone were taken using Zeiss confocal optics in a LSM510 META microscope. Bar in the upper left picture = 50 μm. All the other pictures have the same scale.

**Figure 3:**
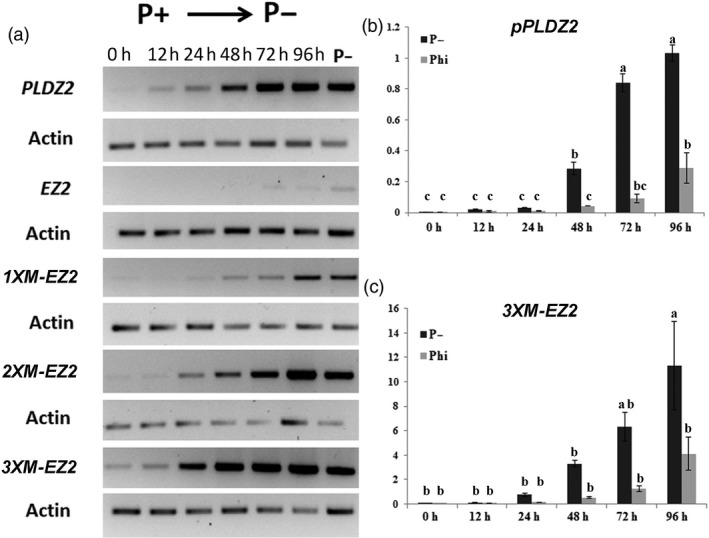
Comparative *GUS* expression analysis among the native *PLDZ2* promoter, *EZ2* native and the *M‐EZ2* enhancer versions upstream of the −46 S minimal 35 promoter in response to Pi deficiency and Phi supply. (a) Semiquantitative RT‐PCR assays of *GUS* transcripts driven by either *pPLDZ2*,* EZ2*,* 1XM‐EZ2*,* 2XM‐EZ2* or *3XM‐EZ2* were carried out for plants grown for 7 dag in P+ (1 mm) and transferred to P− (0 mm) medium for 12 h, 24 h, 48 h, 72 h and 96 h. Transcript levels for plants grown continuously in P− are also indicated. (b) Quantitative reverse transcription PCR analysis for *GUS* expression of plants carrying *pPLDZ2‐GUS::GFP* and (c) *3XM‐EZ2‐GUS::GFP*, grown in P+ (1 mm) for 7dag and transferred to P− (0 mm) or phosphite (Phi, 1 mm) are shown. A time course indicating the transcript level of the reporter gene is shown. Values are reported as a relative quantification [2^(−∆ Cт)^] between the reporter gene and the *ACTIN2* gene for each condition. Data are the mean of two biological replicas, and letters above bars indicate statistically significant differences supported by ANOVA test.

In order to quantify the higher sensitivity of *3XM‐EZ2* promoter in comparison with that of *PLDZ2*, the *GUS* transcripts levels were determinated by a qRT‐PCR. Our results showed that in response to low Pi, the *3XM‐EZ2* promoter induces the expression of the *GUS* transcripts 48.2‐fold 24 h after seedlings were submitted to the stress, whereas for the *PLDZ2* promoter, *GUS* transcript levels at this time point were 7.08‐fold higher than the controls in Pi+ media. This behaviour was similar in the following time points of the treatment; by 48, 72 and 96 h, the *GUS* transcript levels under the *3XM‐EZ2* promoter were, respectively, 207.63, 395.92 and 710.86 while those of the *PLDZ2* promoter in the corresponding times were 69.19, 203.45 and 250.29 (Figure 3b and c). These results correlate well with the qualitative analyses of GFP expression in *PLDZ2‐GUS::GFP* and *3XM‐EZ2‐GUS::GFP* lines, by analysing the expression pattern of the transgene in a similar conditions and time point used for *GUS* q‐RT‐PCR analysis. Our results clearly show a higher GFP expression, especially in the vascular tissue of *3XM‐EZ2‐GUS::GFP* roots, when compared with those of *PLDZ2‐GUS::GFP* (Figure 2).

### *M‐EZ2* displays a high sensitivity to internal and external Pi concentrations

It has been shown that phosphite (Phi), a nonassimilable source of phosphorus for plants, a structural analogue of Pi that it is transported to the cytoplasm via the same transport system as Pi in plant cells (Danova‐Alt *et al*., 2008), is perceived by the plant as Pi altering some of the responses to Pi deficiency, mainly delaying the initial response triggered by external and internal reduction of Pi levels (Ticconi *et al*., 2001; Varadarajan *et al*., 2002). Taking advantage of this knowledge, we tested whether Phi addition to the media could alter the intensity and/or timing of the response of *PLDZ2* and *M‐EZ2* promoters to Pi starvation. For this, *PLDZ2‐GUS::GFP* and *3XM‐EZ2‐GUS::GFP* seedlings were transferred from P+ medium either to Phi+ Pi− or Pi− medium, and samples were taken at different time points (12, 24, 48, 72 and 96 hpt) for RNA extraction and qRT‐PCR evaluation of *GUS* transcripts. Ours results showed that the presence of Phi decreases the *GUS* transcripts at each time point after plants are subjected to low Pi availability. This decrease indirectly shows that Phi delays the response of *PLDZ2* along the time course experiment. For instance, while in plants transferred to Pi− medium there is a clear increase in *GUS* transcripts by 48 hpt (Figure 3b), in plants transferred to Phi+ Pi− medium this increase was observed until 96 hpt and the final level of induction was significantly lower in seedlings transferred to media containing Phi than those transferred into media lacking Pi (Figure 3b). *3XM‐EZ2‐GUS::GFP* seedlings also showed this delay, as plants transferred to media containing Phi had a detectable decrease with respect to the signal detected for seedlings grown in Pi− media. However, the expression is significantly higher than that observed for the *PLDZ2* promoter at all tested time points (Figure 3c). This suggests that three copies of *M‐EZ2* increased the sensitivity of the *3XM‐EZ2* synthetic promoter to Pi availability in such a manner that even in the presence of a molecule that mimics Pi presence, as Phi does, the promoter is still able to strongly respond to Pi scarcity. These results suggest that Phi acts in Arabidopsis plants via the P1BS DNA motif, which is the only regulatory element in the *3XM‐EZ2* synthetic promoter that is known to respond to low phosphate availability and, therefore, it must be by promoting the interaction of PHR1 and SPX1, as previously shown under in vitro binding conditions (Puga *et al*., 2014).

### Reversible behaviour of *M‐EZ2*

**Figure 4:**
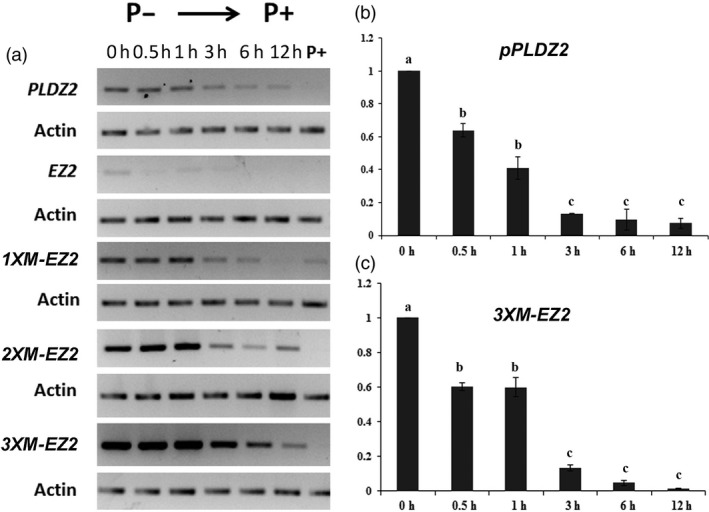
Comparative *GUS* expression analysis among the native *PLDZ2* promoter, *EZ2* native and the *M‐EZ2* enhancer versions upstream of the −46 S minimal 35 promoter in response to Pi supply. (a) Plants grown for 7 dag in medium P− (0 mm) and then transferred to medium P+ (1 mm) were analysed by semiquantitative RT‐PCR assay. *GUS* transcript levels from the *pPLDZ2*,* EZ2* native, *1XM‐EZ2*,* 2XM‐EZ2* and *3XM‐EZ2* constructs are shown at 0.5, 1, 3, 6 and 12 h. GUS transcript levels of plants grown continuously in P+ are also indicated. (b) Real‐time qRT‐PCR analysis of *pPLDZ2::GUS:GFP* and c) *3XM‐EZ2::GUS:GFP* from plants grown 7 dag in P− (0 mm) and transferred to P+ (1 mm) are shown. Values are reported as a relative quantification [2^(−∆∆ Cт)^] in which was considered the sample P− 0 h as calibrator and as an endogenous control *ACTIN*2 gene. Values are the mean of two biological replicas, and letters above bars indicate statistically significant differences resulted from ANOVA test of the data.

To determine whether the expression directed by the EZ2 synthetic promoters is reversible after the transcriptional activation by Pi starvation, we transferred seedling grown in Pi− media for 7 dag into Pi+ media and then determine *GUS* transcript levels. We could clearly observe that the expression of the chimeric promoters was repressed upon transfer from Pi− media into Pi+ media (Figure 4a). This behaviour was quantified by qRT‐PCR (Figure 4b and c) comparing the complete promoter and the *3X* enhancer version. The transcription levels were reduced 36.04% and 39.99% in the first 30 min in *pPLDZ2* and the *3XM‐EZ2* seedlings, respectively. 1, 3, 6 and 12 h post‐treatment the level of transcription for *pPLDZ2* progressively declined in 59.04%, 86.84%, 90.33% and 92.5%. In the case of *3XM‐EZ2* seedlings, this percentage was reduced in 40.16%, 86.83%, 95.56% and 98.82% at the respective time points. These results confirm that the activity of the *M‐EZ2* enhancer is directly regulated by internal levels of Pi.

### Generation and function of a bidirectional Pi‐responsive promoter

**Figure 5:**
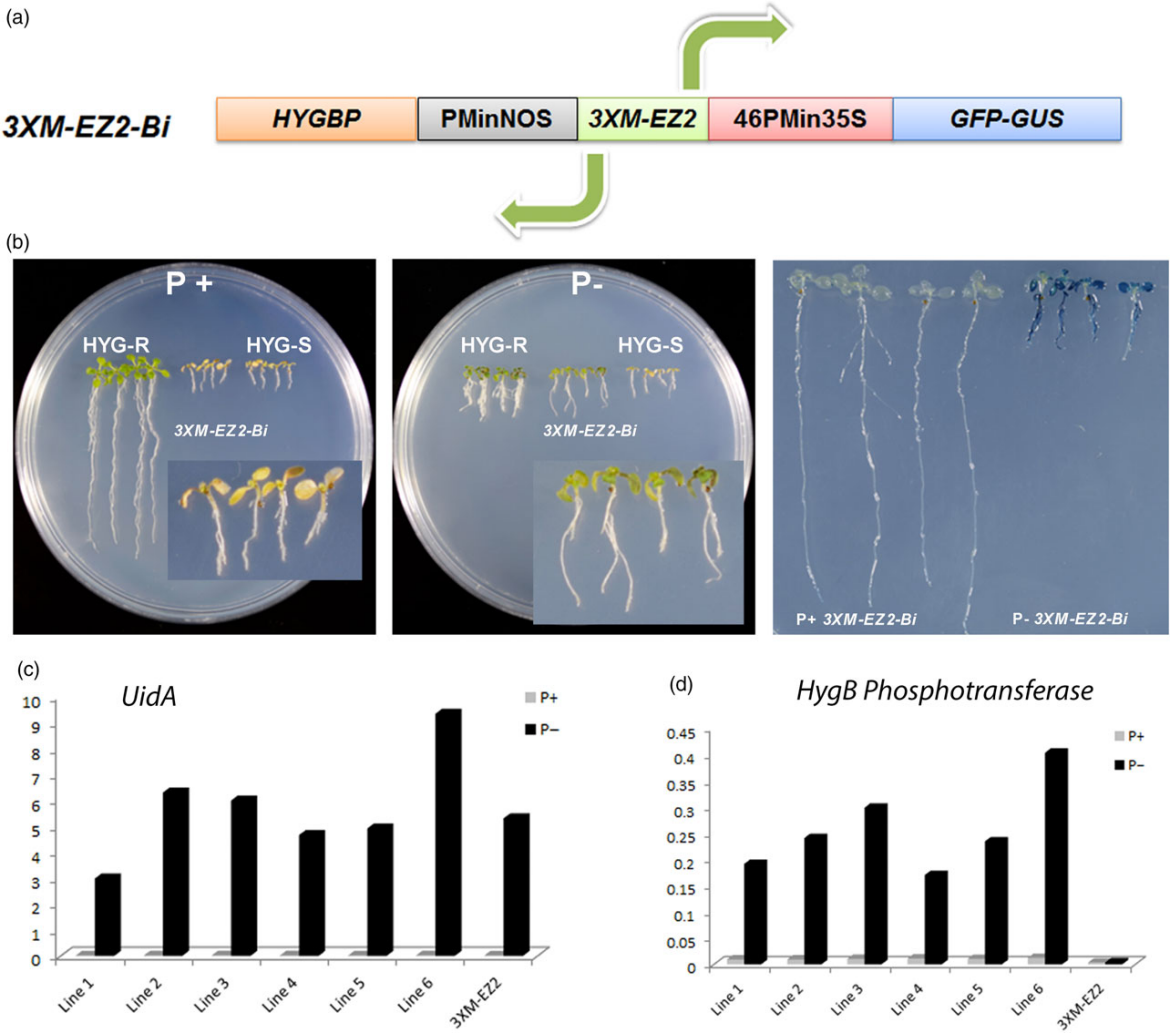
Pi‐starvation responsiveness of bidirectional *3XM‐EZ2* enhancer. (a) Diagram showing the structure of *3XM‐EZ2 Bi*, harbouring at the core the *M‐EZ2* enhancer, at the 5′ end the minimal *NOS* promoter and the *HYG B* phosphotransferase gene in opposite directions, and at the 3′ end the −46 S minimal 35 S promoter followed by the *GFP* and *uidA* genes. (b) Expression analysis of the two reporter genes in Arabidopsis plants. Hygromycin B resistance of Arabidopsis seedlings growing in induction medium without Pi (5 μm) and then transferred to Hyg medium (15 mg/L) with (1 mm) or without (5 μm) Pi. Hygromycin B‐resistant and hygromycin B‐sensitive plants were grown as controls (two left panels). The right panel shows GUS activity of seedlings grown for 10 dag in medium Pi+ (1 mm) and Pi− (0 mm) and then stained and photographed. (c) Quantitative reverse transcription PCR assay of plants carrying *3XM‐EZ2 Bi*. Six independent lines were analysed for (c) *GUS* and (d) *HYG B* phosphotransferase expression, and a representative line carrying *3XM‐EZ2* enhancer was analysed as control. Values are the result of a relative quantification [2^(−∆ Cт)^] between the reporter gene and *ACTIN2* gene for each condition.

In nature, the presence of genes located on opposite DNA strands, which transcription is under the control of a bidirectional promoter has been reported in several cases (Banerjee *et al*., 2013; Dhadi *et al*., 2009; Liu and Han, 2009; Wang *et al*., 2009). The capacity of bidirectional promoters to control the expression of two opposite open reading frames (ORFs) is many times dependent on the presence of enhancer elements that can affect the expression of proximal promoters located upstream and downstream of the enhancer element (Chaturvedi *et al*., 2006). Based on the fact that enhancers can modulate transcription independent of their orientation, genetic engineering strategies have been designed to develop minimal bidirectional promoters that confer a specific spatio‐temporal transcriptional pattern (Mehrotra *et al*., 2011; Venter, 2007). As the P1BS sequence is palindromic (GAATATTC) and *EZ2* acts an enhancer, it is predictable that the activity of the triple enhancer element could act in both orientations. Therefore, we tested the capacity of the *3XM‐EZ2* element to function bidirectionally by fusing a minimum nopaline synthase promoter (PMinNOS) at its 5′ end in opposite direction to 46PMin35S (Figure 5a, Figure S1). The *PMinNOS‐3XM‐EZ2‐46PMin35S* bidirectional promoter recombined in the pBGWFS7 plasmid to generate the *PMinNOS‐3XM‐EZ2‐46PMin35S‐GUS::GFP* transcriptional fusion. Then, the gene coding regions for hygromycin resistance (hygromycin B phosphotransferase or HYGBP) and *GUS‐GFP* were placed downstream of the PMinNOS and 46PMin promoters, respectively. The binary vector carrying the dual transcriptional phusion *HYGBP::PMinNOS‐3XM‐EZ2‐46PMin35S‐GUS::GFP* (Figure 5a) between the T‐DNA borders was used to transform Arabidopsis plants, and the resulting homozygous lines were tested for GUS and HYGBP activity. To test the capacity of *HYGBP::PMinNOS‐3XM‐EZ2‐46PMin35S‐GUS::GFP* (*3XM‐EZ2Bi*) lines to express the Hygromycin resistance gene, seedlings were germinated and grown in solid medium lacking Pi for 6 days and then transferred to either Pi+ or Pi− mediums, both added with 15 mg/L of hygromycin. Seedlings transferred to Pi+/Hyg medium died in a similar manner to hygromycin sensible controls (HYGS in Figure 5b), whereas seedlings transferred to Pi−/Hyg medium were able to survive, developing green cotyledons and leaves similar to that observed for seedlings harbouring the Hygromycin resistance gene under the 35S promoter (HYGR in Figure 5b, left panel). These results correlate well with GUS patterns of *3XM‐EZ2Bi* seedlings transferred to Pi−/Hyg subjected to the staining protocol which show a strong blue after the reaction (Figure 5b, right panel), while the seedlings grown in Pi+ medium do not show detectable blue staining, indicating that they do not express *GUS* gene at all (Figure 5b, right panel).

We performed a quantitative analysis of *GUS* and *HYGBP* transcripts in *3XM‐EZ2 Bi* seedlings grown 7dpg in Pi+ solid medium and then transferred to Pi− medium for 72 h. Seedlings of six independent lines showed a transcriptional *GUS* behaviour similar to the unidirectional *3XM‐EZ2* lines (Figure 5c). In the case of the *HYGBP* gene, transcript levels were also induced in seedlings transferred to Pi− when compared to the control. Our results also evidenced that, although enough to confer hygromycin resistance to plants, the *HYGBP* transcripts levels are 10 times lower than those of *GUS* transcripts, suggesting that either the strength with which *M‐EZ2* enhancer influences the transcription of minimal promoters is orientation dependent or that the nature of the minimal promoter determines the strength of the effect of this enhancer element; in this case, *PMinNOS* can influence the level of induction conferred by the *3XM‐EZ2* enhancer construct (Figure 5d).

## Discussion

In this study, we built a promoter by arranging in tandem three copies of a modified enhancer from the promoter region of the Arabidopsis *PLDZ2* gene, which strongly responds to Pi starvation (*M‐EZ2*; Oropeza‐Aburto *et al*., 2012). We found that the *3XM‐EZ2* promoter is highly sensitive to Pi scarcity, showing that the number of copies of the enhancer drastically influences the strength of the response. It has been previously shown the importance of P1BS as a *cis*‐acting motif in the Pi‐starvation response. However, the presence of P1BS in a given promoter is not enough to confer Pi‐responsiveness to the gene, the specific sequence of the motif and the distribution of one or more motifs in the sequence context define the strength of the transcriptional response to the stress (Bustos *et al*., 2010; Oropeza‐Aburto *et al*., 2012; Ruan *et al*., 2015). We also show that the composition of the *M‐EZ2* element confers an optimal sequence and spatial arrangement to increase the sensitivity to the action of the transcriptional activator PHR, a sensitivity that increases in function of the number of *M‐EZ2* copies.

The P1BS, as a target sequence of PHR1 in a natural or artificial promoter, depends on the amount of this TF and its activity. PHR1 capacity to act on P1BS depends, in turn, on the direct binding among SPX1 and PHR1 proteins, a Pi‐dependent interaction which inhibits PHR1 DNA‐binding. Puga *et al*. (2014) showed that in the presence of Pi the SPX1 binds PHR1, inhibiting the transcriptional activation of PHR1 targets. They also reported that phosphite (Phi) was able to interfere with the interaction between PHR1 and SPX using an *in vitro* binding assay. As the *M‐EZ2* enhancer only contains the P1BS‐binding site as a Pi−responsive element, the observation that the *3XM‐EZ2* is still influenced by Phi provides *in vivo* support of the notion that Phi directly acts by promoting the interaction between PHR1 and SPX1. However under Pi‐starvation conditions, low Pi concentrations decrease the interaction between SPX1 and PHR1 complex, releasing PHR1 which activates its transcriptional targets, among which is *SPX1* gene itself. Indeed, *SPX1* transcription is repressed in the presence of Phi and absence of Pi, in both shoots and roots (Jost *et al*., 2015).

An important observation in this study is that the *3XM‐EZ2* promoter responds to Pi limitation, even in the presence of Phi. This suggests that the sensitivity of the *3XM‐EZ2* promoter to Pi absence is high enough to discriminate among Phi and Pi; in other words, the promoter detects specifically the scarcity of the ion orthophosphate as a signal even in the presence of Phi. However, more and detailed experiments should be performed in the future to fully confirm or discard such hypothesis.

Moreover, we found that the induction of transcription in response to Pi scarcity, driven by the *3XM‐EZ2* promoter, can be reversed by resupplying Pi to the medium. Therefore, our designed promoter behaves as a switch that can be turned On or Off in function of the Pi concentration in the medium. Such features of the *3XM‐EZ2* promoter prompted us to construct a bidirectional version of the promoter by fusing two unidirectional minimal promoters in opposite direction driven by *3XM‐EZ2* cis‐regulatory elements (*3XM‐EZ2Bi*). Our results demonstrated that *3XM‐EZ2Bi* was able to efficiently induce the transcription of two reporter transgenes in Arabidopsis seedlings in response to Pi availability. In the past years, the need for simultaneous multiple gene expression for biotechnological application in plants has led to the generation of bidirectional promoters base on plant cis‐regulatory regions (Chaturvedi *et al*., 2006; Frey *et al*., 2001; Li *et al*., 2004; Mitra *et al*., 2009; Xie *et al*., 2001). The latest attempts have improved the design of bidirectional promoters avoiding gene silencing, and this is the case of a light‐induced ‘natural’ bidirectional promoter of Arabidopsis, which was used to express simultaneously two transgenes (Mitra *et al*., 2009). Although bidirectional promoters occur naturally in plants, very few have been shown to activate the expression of two genes by the same environmental stimuli and with a similar tissue‐specific pattern of expression. Besides Mitra *et al*. (2009) work, to date no natural or designed bidirectional promoters triggered by environmental factors have been reported. To our knowledge, this study describes the first designed bidirectional promoter which can be turned on by phosphate deficiency. Moreover, it behaves in a reversible manner. Therefore, the *3XM‐EZ2Bi* design represents a novel tool for multiple gene expression modulated first by Pi availability and second by the nature of the opposite promoters used. The fact that *3XM‐EZ2* is able to turn On and Off in a high sensitive fashion, depending on the Pi concentrations in plant roots, points to very promising potential uses of this tool not only for *in vitro* research and biotechnology applications, but also for crop improvement approaches to allow plants grow in soils with low phosphate amounts and other stresses, as the bidirectionality of the promoter will allow the expression of genes to cope with more than one abiotic stress.

## Experimental procedures

### Plant material and growth conditions

*Arabidopsis thaliana* transgenic lines were generated in Col0 ecotype background. Seeds were disinfected with 20% (v/v) bleach in water, followed by several rinses of sterile distilled water. The medium used for germination and all other experiments was MS 0.1X supplemented with NaH_2_PO_4_ (1 mm) or without NaH_2_PO_4_ (0 mm), 0.5% sucrose and 10 g/L agar.

### Protein extraction and fluorometric GUS assays

GUS activity was determined in plantlets grown 10dag under limiting (0 mm) or sufficient (1 mm) phosphate. The plantlets were ground in protein extraction buffer containing 50 mm KPO_4_, pH 7.0, 10 mm EDTA, 0.1% Triton X‐100, 0.1% Sarkosyl and 10 mm β‐mercaptoethanol, and protein was quantified by Bio‐Rad Protein Assay. One μg of plant protein extract from the phosphate‐limiting or phosphate‐sufficient conditions was incubated with 2 mm of 4‐methylumbelliferyl‐b‐D‐glucuronide in protein extraction buffer for 90 min and 240 min, respectively. GUS activity was measured fluorometrically using a high‐performance multilabel plate reader TECAN Infinite M1000.

### Histochemical GUS

Seedlings grown for 10 dag on solid medium with (1 mm) or without (0 mm) phosphate were incubated in GUS reaction buffer (0.5 mg/mL of 5‐bromo‐4‐chloro‐3‐indolyl‐b‐D‐glucuronide in 100 mm sodium phosphate, pH 7.0) overnight at 37 °C. After, tissues were cleared (Malamy and Benfey, 1997) and representative plants were photographed using Nomarski optics in a Leica DMR microscope.

### Real‐time quantitative analysis

Plants carrying the *pPLDZ2‐GUS::GFP* and *3XM‐EZ2‐GUS::GFP* were grown for 7 dag in Pi‐sufficient medium and then transferred to liquid medium without Pi for 12, 24, 48, 72 and 96 h, or liquid medium with Phi (1 mm); plants were then collected, frozen and ground to isolate total RNA using the TRIzol reagent method (Invitrogen). The same lines were grown in Pi‐limiting medium and then transferred to Pi‐sufficient liquid medium for 0.5, 1, 3, 6 and 12 h. cDNA was synthesized with SuperScript III reverse transcriptase (Invitrogen) using 30 μg of total RNA for each sample. The qPCR was performed in a Real‐time PCR ABI PRISM 7500 sequence detection system (Applied Biosystems), using SYBR Green PCR Master Mix (Applied Biosystems) and specific primers (Table S2). The PCR conditions were as follows: 10 min at 95 °C and 40 cycles at 95 °C for 30 s, 60 °C for 30 s and 72 °C for 40 s. Relative transcript abundance was determined using the *ACTIN2* transcript as control. At least three independent PCRs were performed for each sample. The same procedure for qRT‐PCR assay was used to analyse plants carrying the *3XM‐EZ2Bi* grown in medium P+ (1 mm) for 7 dag and transferred for 72 h to P− (0 mm) liquid medium.

### Semiquantitative RT‐PCR assay

RNA was isolated from plants carrying the *EZ2‐GUS::GFP*,* pPLDZ2‐GUS::GFP*,* 1XM‐EZ2‐GUS::GFP*,* 2XM‐EZ2‐GUS::GFP* and *3XM‐EZ2‐GUS::GFP* constructs. Plants were subjected to transfer experiments from Pi sufficient to Pi limiting, and Pi limiting to Pi sufficient as in real‐time assay. cDNA synthesis was performed using 100 ng of total RNA with SuperScript III (Invitrogen) following the manufacturer instructions. The PCR amplification conditions for *GUS* transcripts were 94 °C for 3 min and 26 cycles at 94 °C for 30 s, 58 °C for 30 s and a final extension step at 72 °C for 40 s. PCR amplification for *ACTIN2* were 94 °C for 3 min and 25 cycles of 94 °C for 30 s, 60 °C for 30 s and 72 °C for 30 s.

### The *1X*,* 2X* and *3XM‐EZ2* enhancer element construct

We used the previously reported construct *EZ2P1BS4(2X)‐GUS::GFP* in plasmid pKGWFS7 (Oropeza‐Aburto *et al*., 2012) as a backbone to generate new constructs containing two and three times this modified enhancer element (*M‐EZ2*) driving *GUS‐GFP* expression.

We designed single‐stranded oligonucleotides containing the sequence of the DNA chain with one (*M‐EZ2*) or two modified enhancer elements and *Hind* III in both extreme sites (Table S2). We then synthesized the complementary chain with the DNA polymerase Klenow Fragment using a reverse specific primer (Table S2). These double‐stranded fragments were restricted with *Hind* III and cloned in the *Hind* III site of the backbone plasmid pKGWFS7 in which the modified enhancer element *EZ2P1BS4(2X)‐GUS::GFP* had been cloned previously (Oropeza‐Aburto *et al*., 2012).

These constructs were introduced in *Agrobacterium tumefaciens* and then used to transform *Arabidopsis thaliana* by the floral dip method (Martinez‐Trujillo *et al*., 2004).

### The *3XM‐EZ2* bidirectional hygromycin and GUS‐GFP construct

A bidirectional enhancer *3XM‐EZ2* containing the *EZ2* enhancer element three times, the −46 minimum promoter of 35S at the 3′ and the minimum promoter of the nopaline synthase gene in opposite direction at the 5′ end, was synthesized (Figure S1).

This fragment carrying the *3XM‐EZ2* bidirectional enhancer was cloned by recombination in pDONR221 plasmid using a GATEWAY BP kit (Invitrogen), and then subcloned in the destiny plasmid pBGWFS7 using the GATEWAY LR kit (Invitrogen).

A DNA fragment containing the hygromycin B phosphotransferase gene and the NOS terminator was obtained by XbaI restriction from plasmid pWRG1515 and isolated. This fragment was cloned in the unique Xba I site of plasmid pBGWFS7 carrying the *3XM‐ EZ2* bidirectional enhancer, verifying that it had been cloned in the correct orientation.

This construction was introduced in *Agrobacterium tumefaciens* by electroporation, and this strain was used for Arabidopsis transformation by the floral dip method (Martinez‐Trujillo *et al*., 2004).

## Conflict of interest

The authors declare no conflict of interests.

## Supporting information


**Figure S1.** Sequence and arrangement of the *3XM‐EZ2* bidirectional enhancer element. In Italics is represented minimum prom NOS at 5′ in opposite direction, and at the 3′ extreme the minimum promoter −46 S. In box, restriction sites EcoR1, Spe I and Bgl II. In bold enhancer *3XM‐EZ2*. Underlined P1BS sites. Lowercase, attB1 and attB2 recombination sites.
**Table S1.** GUS activity of pPLDZ2, EZ2, 1XM‐EZ2, 2XM‐EZ2 and 3XM‐EZ2 constructions. p35S activity is shown as control. Values are the mean of 4 technical replicas, and sample 1 and 2 are biological replicas.
**Table S2.** Sequence of oligonucleotides used for synthesize DNA fragments to generate the in‐tandem enhancers and primers used for Real‐Time PCR and RT‐PCR assays.

## References

[pbi12653-bib-0001] Acevedo‐Hernández, G. , Oropeza‐Aburto, A. and Herrera‐Estrella, L. (2012) A specific variant of the PHR1 binding site is highly enriched in the Arabidopsis phosphate‐responsive phospholipase DZ2 coexpression network. Plant Signaling & Behavior, 7, 914–917. 22836502 10.4161/psb.20749 PMC3474684

[pbi12653-bib-0002] Baker, A. , Ceasar, S.A. , Palmer, A.J. , Paterson, J.B. , Qi, W. , Muench, S.P. and Baldwin, S.A. (2015) Replace, reuse, recycle: improving the sustainable use of phosphorus by plants. J. Exp. Bot. 66, 3523–3540. 25944926 10.1093/jxb/erv210

[pbi12653-bib-0003] Banerjee, J. , Sahoo, D.K. , Dey, N. , Houtz, R.L. and Maiti, I.B. (2013) An intergenic region shared by At4g35985 and At4g35987 in Arabidopsis thaliana is a tissue specific and stress inducible bidirectional promoter analyzed in transgenic Arabidopsis and tobacco plants. PLoS ONE, 8, e79622. 24260266 10.1371/journal.pone.0079622 PMC3834115

[pbi12653-bib-0004] Bari, R. , Pant, B.D. , Stitt, M. and Scheible, W.‐R. (2006) PHO2, microRNA399, and PHR1 define a phosphate‐signaling pathway in plants. Plant Physiol. 141, 988–999. 16679424 10.1104/pp.106.079707 PMC1489890

[pbi12653-bib-0005] Bustos, R. , Castrillo, G. , Linhares, F. , Puga, M.I. , Rubio, V. , Pérez‐Pérez, J. , Solano, R. *et al* (2010) A central regulatory system largely controls transcriptional activation and repression responses to phosphate starvation in Arabidopsis. PLoS Genet. 6, e1001102. 20838596 10.1371/journal.pgen.1001102 PMC2936532

[pbi12653-bib-0006] Chaturvedi, C.P. , Sawant, S.V. , Kiran, K. , Mehrotra, R. , Lodhi, N. , Ansari, S.A. and Tuli, R. (2006) Analysis of polarity in the expression from a multifactorial bidirectional promoter designed for high‐level expression of transgenes in plants. J. Biotechnol. 123, 1–12. 16324763 10.1016/j.jbiotec.2005.10.014

[pbi12653-bib-0007] Chiou, T.‐J. and Lin, S.‐I. (2011) Signaling network in sensing phosphate availability in plants. Annu. Rev. Plant Biol. 62, 185–206. 21370979 10.1146/annurev-arplant-042110-103849

[pbi12653-bib-0008] Danova‐Alt, R. , Dijkema, C. , De Waard, P. and Koeck, M. (2008) Transport and compartmentation of phosphite in higher plant cells–kinetic and 31P nuclear magnetic resonance studies. Plant, Cell Environ. 31, 1510–1521. 18657056 10.1111/j.1365-3040.2008.01861.x

[pbi12653-bib-0009] Dhadi, S.R. , Krom, N. and Ramakrishna, W. (2009) Genome‐wide comparative analysis of putative bidirectional promoters from rice, Arabidopsis and Populus. Gene, 429, 65–73. 18973799 10.1016/j.gene.2008.09.034

[pbi12653-bib-0010] Franco‐Zorrilla, J.M. , González, E. , Bustos, R. , Linhares, F. , Leyva, A. and Paz‐Ares, J. (2004) The transcriptional control of plant responses to phosphate limitation. J. Exp. Bot. 55, 285–293. 14718495 10.1093/jxb/erh009

[pbi12653-bib-0011] Frey, P.M. , Schärer‐Hernández, N.G. , Fütterer, J. , Potrykus, I. and Puonti‐Kaerlas, J. (2001) Simultaneous analysis of the bidirectional African cassava mosaic virus promoter activity using two different luciferase genes. Virus Genes, 22, 231–242. 11324760 10.1023/a:1008183827072

[pbi12653-bib-0012] Jost, R. , Pharmawati, M. , Lapis‐Gaza, H.R. , Rossig, C. , Berkowitz, O. , Lambers, H. and Finnegan, P.M. (2015) Differentiating phosphate‐dependent and phosphate‐independent systemic phosphate‐starvation response networks in Arabidopsis thaliana through the application of phosphite. J. Exp. Bot. 66, 2501–2514. 25697796 10.1093/jxb/erv025 PMC4986860

[pbi12653-bib-0013] Li, Z.T. , Jayasankar, S. and Gray, D. (2004) Bi‐directional duplex promoters with duplicated enhancers significantly increase transgene expression in grape and tobacco. Transgenic Res. 13, 143–154. 15198202 10.1023/b:trag.0000026074.11859.77

[pbi12653-bib-0014] Lin, W.‐Y. , Lin, S.‐I. and Chiou, T.‐J. (2009) Molecular regulators of phosphate homeostasis in plants. J. Exp. Bot. 60, 1427–1438. 19168668 10.1093/jxb/ern303

[pbi12653-bib-0015] Liu, X. and Han, B. (2009) Evolutionary conservation of neighbouring gene pairs in plants. Gene, 437, 71–79. 19264115 10.1016/j.gene.2009.02.012

[pbi12653-bib-0016] Lundmark, M. , Kørner, C.J. and Nielsen, T.H. (2010) Global analysis of microRNA in Arabidopsis in response to phosphate starvation as studied by locked nucleic acid‐based microarrays. Physiol. Plant. 140, 57–68. 20487378 10.1111/j.1399-3054.2010.01384.x

[pbi12653-bib-0017] Lynch, J. (1995) Root architecture and plant productivity. Plant Physiol. 109, 7. 12228579 10.1104/pp.109.1.7 PMC157559

[pbi12653-bib-0018] Malamy, J.E. and Benfey, P.N. (1997) Organization and cell differentiation in lateral roots of Arabidopsis thaliana. Development, 124, 33–44. 9006065 10.1242/dev.124.1.33

[pbi12653-bib-0019] Martinez‐Trujillo, M. , Limones‐Briones, V. , Cabrera‐Ponce, J.L. and Herrera‐Estrella, L. (2004) Improving transformation efficiency of Arabidopsis thaliana by modifying the floral dip method. Plant Mol. Biol. Rep. 22, 63–70.

[pbi12653-bib-0020] Mehrotra, R. , Gupta, G. , Sethi, R. , Bhalothia, P. , Kumar, N. and Mehrotra, S. (2011) Designer promoter: an artwork of cis engineering. Plant Mol. Biol. 75, 527–536. 21327513 10.1007/s11103-011-9755-3

[pbi12653-bib-0021] Mitra, A. , Han, J. , Zhang, Z.J. and Mitra, A. (2009) The intergenic region of Arabidopsis thaliana cab1 and cab2 divergent genes functions as a bidirectional promoter. Planta, 229, 1015–1022. 19169705 10.1007/s00425-008-0859-1

[pbi12653-bib-0022] Müller, R. , Morant, M. , Jarmer, H. , Nilsson, L. and Nielsen, T.H. (2007) Genome‐wide analysis of the Arabidopsis leaf transcriptome reveals interaction of phosphate and sugar metabolism. Plant Physiol. 143, 156–171. 17085508 10.1104/pp.106.090167 PMC1761981

[pbi12653-bib-0023] Nilsson, L. , Müller, R. and Nielsen, T.H. (2007) Increased expression of the MYB‐related transcription factor, PHR1, leads to enhanced phosphate uptake in Arabidopsis thaliana. Plant, Cell Environ. 30, 1499–1512. 17927693 10.1111/j.1365-3040.2007.01734.x

[pbi12653-bib-0024] Oropeza‐Aburto, A. , Cruz‐Ramírez, A. , Acevedo‐Hernández, G.J. , Pérez‐Torres, C.‐A. , Caballero‐Pérez, J. and Herrera‐Estrella, L. (2012) Functional analysis of the Arabidopsis PLDZ2 promoter reveals an evolutionarily conserved low‐Pi‐responsive transcriptional enhancer element. J. Exp. Bot. 63, 2189–2202. 22210906 10.1093/jxb/err446 PMC3295404

[pbi12653-bib-0025] Plaxton, W.C. (2004) Plant Response to Stress: Biochemical Adaptations to Phosphate Deficiency. Encyclopedia of Plant and Crop Science, pp. 976–980. New York: Marcel Dekker.

[pbi12653-bib-0026] Puga, M.I. , Mateos, I. , Charukesi, R. , Wang, Z. , Franco‐Zorrilla, J.M. , de Lorenzo, L. , Irigoyen, M.L. *et al* (2014) SPX1 is a phosphate‐dependent inhibitor of PHOSPHATE STARVATION RESPONSE 1 in Arabidopsis. Proc. Natl. Acad. Sci. 111, 14947–14952. 25271326 10.1073/pnas.1404654111 PMC4205628

[pbi12653-bib-0027] Raghothama, K. (1999) Phosphate acquisition. Annu. Rev. Plant Biol. 50, 665–693. 10.1146/annurev.arplant.50.1.665 15012223

[pbi12653-bib-0028] Rouached, H. , Arpat, A.B. and Poirier, Y. (2010) Regulation of phosphate starvation responses in plants: signaling players and cross‐talks. Molecul. Plant, 3, 288–299. 10.1093/mp/ssp120 20142416

[pbi12653-bib-0029] Ruan, W. , Guo, M. , Cai, L. , Hu, H. , Li, C. , Liu, Y. , Wu, Z. *et al* (2015) Genetic manipulation of a high‐affinity PHR1 target cis‐element to improve phosphorous uptake in Oryza sativa L. Plant Mol. Biol. 87, 429–440. 25657119 10.1007/s11103-015-0289-y

[pbi12653-bib-0030] Rubio, V. , Linhares, F. , Solano, R. , Martín, A.C. , Iglesias, J. , Leyva, A. and Paz‐Ares, J. (2001) A conserved MYB transcription factor involved in phosphate starvation signaling both in vascular plants and in unicellular algae. Genes Dev. 15, 2122–2133. 11511543 10.1101/gad.204401 PMC312755

[pbi12653-bib-0031] Shen, J. , Yuan, L. , Zhang, J. , Li, H. , Bai, Z. , Chen, X. , Zhang, W. *et al* (2011) Phosphorus dynamics: from soil to plant. Plant Physiol. 156, 997–1005. 21571668 10.1104/pp.111.175232 PMC3135930

[pbi12653-bib-0032] Sobkowiak, L. , Bielewicz, D. , Malecka, E. , Jakobsen, I. , Albrechtsen, M. , Szweykowska‐Kulinska, Z. and Pacak, A.M. (2012) The role of the P1BS element containing promoter‐driven genes in Pi transport and homeostasis in plants. Front. Plant Sci. doi: 10.3389/fpls.2012.00058. 10.3389/fpls.2012.00058 PMC3355690 22639653

[pbi12653-bib-0033] Ticconi, C.A. , Delatorre, C.A. and Abel, S. (2001) Attenuation of phosphate starvation responses by phosphite in Arabidopsis. Plant Physiol. 127, 963–972. 11706178 PMC129267

[pbi12653-bib-0034] Vance, C.P. , Uhde‐Stone, C. and Allan, D.L. (2003) Phosphorus acquisition and use: critical adaptations by plants for securing a nonrenewable resource. New Phytol. 157, 423–447. 10.1046/j.1469-8137.2003.00695.x 33873400

[pbi12653-bib-0035] Varadarajan, D.K. , Karthikeyan, A.S. , Matilda, P.D. and Raghothama, K.G. (2002) Phosphite, an analog of phosphate, suppresses the coordinated expression of genes under phosphate starvation. Plant Physiol. 129, 1232–1240. 12114577 10.1104/pp.010835 PMC166517

[pbi12653-bib-0036] Venter, M. (2007) Synthetic promoters: genetic control through cis engineering. Trends Plant Sci. 12, 118–124. 17292658 10.1016/j.tplants.2007.01.002

[pbi12653-bib-0037] Wang, Q. , Wan, L. , Li, D. , Zhu, L. , Qian, M. and Deng, M. (2009) Searching for bidirectional promoters in Arabidopsis thaliana. BMC Bioinform. 10, 1. 10.1186/1471-2105-10-S1-S29 PMC2648788 19208129

[pbi12653-bib-0038] Williamson, L.C. , Ribrioux, S.P. , Fitter, A.H. and Leyser, H.O. (2001) Phosphate availability regulates root system architecture in Arabidopsis. Plant Physiol. 126, 875–882. 11402214 10.1104/pp.126.2.875 PMC111176

[pbi12653-bib-0039] Wykoff, D.D. , Grossman, A.R. , Weeks, D.P. , Usuda, H. and Shimogawara, K. (1999) Psr1, a nuclear localized protein that regulates phosphorus metabolism in Chlamydomonas. Proc. Natl. Acad. Sci. 96, 15336–15341. 10611385 10.1073/pnas.96.26.15336 PMC24820

[pbi12653-bib-0040] Xie, M. , He, Y. and Gan, S. (2001) Bidirectionalization of polar promoters in plants. Nat. Biotechnol. 19, 677–679. 11433282 10.1038/90296

