# Supplementary material for: A phosphate starvation‐driven bidirectional promoter as a potential tool for crop improvement and in vitro plant biotechnology
Source: Plant Biotechnol J. 2016 Dec 27;15(5):558–67. doi: 10.1111/pbi.12653 (PMC5398999; doi:10.1111/pbi.12653)
Supplement: Supplementary file 1 — Figure S1. Sequence and arrangement of the 3XM‐EZ2 bidirectional enhancer element. In Italics is represented minimum prom NOS at 5′ in opposite direction, and at the 3′ extreme the minimum promoter −46 S. In box, restriction sites EcoR1, Spe I and Bgl II. In bold enhancer 3XM‐EZ2. Underlined P1BS sites. Lowercase, attB1 and attB2 recombination sites. Table S1. GUS activity of pPLDZ2, EZ2, 1XM‐EZ2, 2XM‐EZ2 and 3XM‐EZ2 constructions. p35S activity is shown as control. Values are the mean of 4 technical replicas, and sample 1 and 2 are biological replicas. Table S2. Sequence of oligonucleotides used for synthesize DNA fragments to generate the in‐tandem enhancers and primers used for Real‐Time PCR and RT‐PCR assays. [file PBI-15-558-s001.pdf]

Supplementary material for online publication only

ggggacaagtttgtacaaaaaagcaggctac**GAATTC***GACTCTAATTGGATACCGAGGGGAATTTAT*
GGAACGTCAGTGGAGCATTTTTGACAAGAAATATTTGCTAGCTGATAGTGACCTTAGG
*CGACTTTTGAACGCGCA***GAATTAAATAAGTGTGTTTGGGAATATTCTGATCTG**
GCAAAGCACCAAAAAATTGGAATATTCTTGAATTAAATAAGTGTGTTTGGG
AATATTCTGATCTGGCAAAGCACCAAAAAATTGGAATATTCTTGAATTAAA
TAAGTGTGTTTGGGAATATTCTGATCTGGCAAAGCACCAAAAAATTGGAAT
ATTCTTACTAGT*GGATCTTCGCAAGACCCTTCCTCTATATAAGGAAGTTCATTTCAT*
*TTGGAGAGGACACGCTGAAGCT***AGATCTGAATTC**tacccagctttcttgtacaaagtggtcccc

Supplementary Figure 1. Sequence and arrangement of the *3XM-EZ2* bidirectional enhancer element. In Italics is represented minimum prom NOS at 5’ in opposite direction, and at the 3’ extreme the minimum promoter -46S. In box, restriction sites EcoR1, Spe I and Bgl II. In bold enhancer *3XM-EZ2*. Underlined P1BS sites. Lowercase, attB1 and attB2 recombination sites.

Supplementary Table 1. GUS activity of pPLDZ2, EZ2, 1XM-EZ2, 2XM-EZ2 and 3XM-EZ2 constructions. p35S activity is shown as control. Values are the mean of 4 technical replicas, and sample 1 and 2 are biological replicas.

Fluorometric GUS activity (pmolMU µg protein^{-1}min^{-1})								
	P+				P-			
	Sample 1	S.D.	Sample 2	S.D.	Sample 1	S.D.	Sample 2	S.D.
1XM- EZ2	0.761588	0.669508	0.216231	0.139591	2.851689	0.22148	5.000746	0.313199
	7.111759	0.491729	7.408529	0.195737	74.58814	1.433836	55.04474	1.028059
	3.139167	0.503757	2.443618	0.217075	12.82799	0.46096	19.36092	0.162966
	2.736759	0.175333	2.332354	0.078024	23.82182	0.410421	27.68158	0.537599
	2.92366	0.101277	2.769014	0.141929	30.19803	0.410471	22.044	0.434776
	3.320347	0.17992	3.457594	0.089528	37.23785	0.215508	33.83594	1.133751
	0.32803	0.082044	0.413568	0.123985	6.435193	0.261342	22.47726	0.258271
	0.278444	0.179231	0.308601	0.117462	10.64913	0.24391	19.93512	0.21791
2XM- EZ2	1.182432	0.127118	1.633277	0.073326	219.3495	7.19405	483.9448	11.81131
	1.308304	0.227924	1.410749	0.351042	61.8855	1.217091	89.60626	1.78614
	3.87342	0.081063	2.90757	0.076657	30.10402	1.524155	41.35794	1.181317
	0.350915	0.165794	0.541627	0.175257	45.72655	1.777953	37.88143	0.362203
	1.565769	0.164133	1.557702	0.033149	19.49729	1.015673	19.57494	0.41217
	0.669409	0.08948	0.873321	0.074912	161.5434	6.279294	104.7025	0.470147
	3.268854	0.180526	3.776691	0.376321	454.5778	17.47772	491.8583	4.997988
	3.253596	1.367244	2.764815	0.133127	79.69795	2.427632	116.4422	0.449889
3XM- EZ2	5.193167	1.272191	5.504439	0.209246	649.2429	29.89489	870.1454	6.879983
	10.74106	0.12102	13.70651	0.333787	100.928	3.627475	100.9858	0.801547
	8.601242	0.535634	10.49874	0.957515	368.0705	10.72441	357	10.09174
	5.622276	0.247783	6.982366	0.151365	191.8649	9.109379	376.4444	3.725105
	21.98371	0.232756	24.63982	0.900851	875.2082	32.80588	821.7092	6.588164
	7.334895	0.794009	13.02633	0.626562	399.5976	10.37932	389.3221	9.487666
	19.53627	0.662655	21.43479	0.980045	551.6329	15.4069	794.2837	30.15096
	13.13463	0.47073	9.962046	2.266597	32.19897	0.531601	50.58638	1.495739
pPLDZ2	1.462186	0.083322	1.469969	0.149412	24.60793	0.628366	25.7016	0.714082
	3.92806	0.229748	3.351064	0.283603	114.0147	3.78786	202.438	4.442757
	0.576013	0.475411	0.521664	0.303709	26.52496	0.451341	23.3282	0.519141
	0.431528	0.169295	0.608932	0.144082	36.37225	0.464872	30.9159	1.437448
	0.458498	0.209858	0.31998	0.096757	84.442	0.891433	128.7803	4.020476
	0.91507	1.301239	0.205563	0.093247	16.09178	0.287077	4.284456	0.221954
	5.523173	0.532629	6.610982	0.051988	51.25624	0.949978	27.13489	1.344033
	0.616469	0.152438	0.665171	0.083876	16.51836	1.431342	20.32291	0.881851
EZ2	0.335205	0.271096	0.16807	0.089235	3.561674	0.349284	1.309996	0.121424
	0.344837	0.074545	0.837766	0.790539	3.340675	0.286524	2.876853	0.939352
	0.470057	0.167768	0.26762	0.06157	4.471366	0.177939	2.804932	0.226971
	0.366028	0.202953	0.473183	0.453243	1.531571	0.263471	1.402466	0.286398
min 35S	951.1493	16.15977	876.4949	11.57581	734.743	13.87392	714.2404	17.67162

Supplementary Table 2. Sequence of oligonucleotides used for synthesize DNA fragments to generate the in-tandem enhancers and primers used for Real Time PCR and RT-PCR assays.

Oligonucleotides used to generate the in-tandem enhancers

Name	Sequence
Primer A	5′ CCCAAGCTTGAATTAAATAAGTGTGTTTGGGAATATTCTGATCTGGCAAAGCACCAAAAAATTGG AATATTCTTGAATTAAATAAGTGTGTTTGGGAATATTCTGATCTGGCAAAGCACCAAAAAATTGGAATATT CTTAAGCTTCGC 3′
Primer B	5′ CCCAAGCTTGAATTAAATAAGTGTGTTTGGGAATATTCTGATCTGGCAAAGCACCAAAAAA TTGGAATATTCTTAAGCTTCGC 3′
Reverse	5′ GCGAAGCTTA 3′

Oligonucleotides used for Real Time and RT-PCR analysis

Name	Sequence
Hyg fwd	5′GACTGGAGCGAGGCGATGTTCG 3′
Hyg rev	5′ATTTGTGTACGCCCGACAGTCC 3′
GUS fwd	5′ TGCTGTCGGCTTTAACCTCT 3′
GUS rev	5′ GGCACAGCACATCAAAGAGA 3′
ACTIN fwd	5′ GAATTGTCTCGTTGTCCTCCTC 3′
ACTIN rev	5′ TGCAAATCCAGCCTTCACCATAC 3′
